# Genome-Wide Analysis of Cyclophilin Proteins in 21 Oomycetes

**DOI:** 10.3390/pathogens9010024

**Published:** 2019-12-26

**Authors:** Yan Zhang, Kyle Fletcher, Rongkui Han, Richard Michelmore, Ruiwu Yang

**Affiliations:** 1 College of Life Science, Sichuan Agricultural University, Ya’an 625014, China; m15183506429@163.com; 2 Genome Center, University of California, Davis, CA 95616, USA; kfletcher@ucdavis.edu (K.F.); rkbhan@ucdavis.edu (R.H.); rwmichelmore@ucdavis.edu (R.M.)

**Keywords:** cyclophilin, *Phytophthora*, *Plasmopara*, oomycete, effector

## Abstract

Cyclophilins (CYPs), a highly-conserved family of proteins, belong to a subgroup of immunophilins. Ubiquitous in eukaryotes and prokaryotes, CYPs have peptidyl-prolyl cis–trans isomerase (PPIase) activity and have been implicated as virulence factors in plant pathogenesis by oomycetes. We identified 16 CYP orthogroups from 21 diverse oomycetes. Each species was found to encode 15 to 35 CYP genes. Three of these orthogroups contained proteins with signal peptides at the N-terminal end, suggesting a role in secretion. Multidomain analysis revealed five conserved motifs of the CYP domain of oomycetes shared with other eukaryotic PPIases. Expression analysis of CYP proteins in different asexual life stages of the hemibiotrophic *Phytophthora infestans* and the biotrophic *Plasmopara halstedii* demonstrated distinct expression profiles between life stages. In addition to providing detailed comparative information on the CYPs in multiple oomycetes, this study identified candidate CYP effectors that could be the foundation for future studies of virulence.

## 1. Introduction

Cyclophilins (CYPs), a highly-conserved protein family, are ubiquitous in eukaryotes and prokaryotes. CYPs belong to the peptidyl-prolyl cis–trans isomerase (PPIase) family that catalyze the trans–cis isomerization of peptide bonds with proline residues, a rate-limiting step in many protein folding processes [1,2,3]. As such, CYPs have diverse roles including protein folding [4], signaling [5], transcriptional regulation [6], pre-mRNA splicing [7], cell cycle regulation [8], hormone signaling [9], vesicular import pathway [10], and have a role in both meiosis and mitosis [11,12]. In addition, CYPs are typically present in all cellular compartments; for example, CypA is located in the cytoplasm and nuclei, CypC is located in the vacuole, and CypD is located in mitochondria [13].

In mammals and plants, CYPs, FK506 binding proteins, and parvulins are the three immunophilin protein superfamilies. Immunophilins have been demonstrated to bind to cyclosporin A (CsA), an immunosuppressant molecule of fungal origin [14,15,16]. This recognition form contains complexes that affect dendritic and T cells [1,17]. In fungi, CYPs have been identified as targets for CsA. Binding of CsA to Cyp1 compromises the immune response by inhibiting calmodulin-dependent phosphoprotein phosphatase calcineurin [18]. In both wound-infecting *Cryphonectria parasitica* and appressorium-forming *Magnaporthe grisea*, mutant Δcyp1 strains were less virulent on their respective hosts [19,20]. Phenotypically, this was observed as inhibiting appressorium development in *M. grisea* [19]. These data suggest an important role for Cyp1 in pathogenesis. It is yet to be investigated whether CYP homologs have similar roles in oomycetes, phylogenetically distinct organisms with similar pathogenic lifestyles.

In the oomycetes, CYPs have previously only been cataloged in *Phytophthora* spp. [17], and virulence activities of pathogen CYPs have yet to be demonstrated in oomycetes. However, previous studies have demonstrated that host CYPs are targets for pathogen effectors that interact with host proteins to modulate defense responses and facilitate successful infection. During soybean infection by *Phytophthora sojae*, the PsAvr3b effector is delivered into host cells, where it is activated by direct interaction with the host cyclophilin GmCYP1; this activation is required for both virulence and avirulence activities [21,22,23].

This study identified oomycete candidate effectors by annotating cyclophilins from 21 diverse oomycete species including nine genera across four families (Saprolegniales, Pythiales, Albuginales, and Peronosporales). These oomycetes present widely divergent life styles, including saprotrophs, necrotrophs, hemibiotrophs, and biotrophs, with broad or narrow host ranges of plants or animals. Evidence of CYP gene expression in *Phytophthora infestans* and *Plasmopara halstedii* were further characterized by analyzing transcriptional data obtained during asexual development. This analysis revealed 16 distinct CYP orthogroups, of which half were ubiquitous in all oomycetes; the number of CYPs annotated in each species varied from 15 to 35. This study provides a deeper understanding of the prevalence and possible functions of CYPs in oomycetes.

## 2. Results and Discussion

### 2.1. Structure Analysis

The number of CYPs identified for each oomycete varied between species (Table 1 and Supplementary Table S1). These proteins were clustered into 16 orthogroups, 13 of which were ubiquitous across the 9 genera surveyed. Orthogroups oomcCYP14 and oomcCYP15 were absent in every biotrophic species (Table 1). Orthogroup oomcCYP12 was absent from *Albugo* spp., an oomycete genus that adapted to biotrophy independently from the downy mildews [24]. Manual curation identified 43 of 472 proteins across 17 species that were likely misannotated (Table 1, Supplementary Table S2). Conserved domain analysis identified partial/low scoring CYP domains, whereas orthologs had higher confidence CYP domains. Investigating the annotation identified high scoring CYP domains split across multiple reading frames, implying that a splice site may have not been predicted; this intron position is not conserved across all oomycete species.

**Figure 1:**
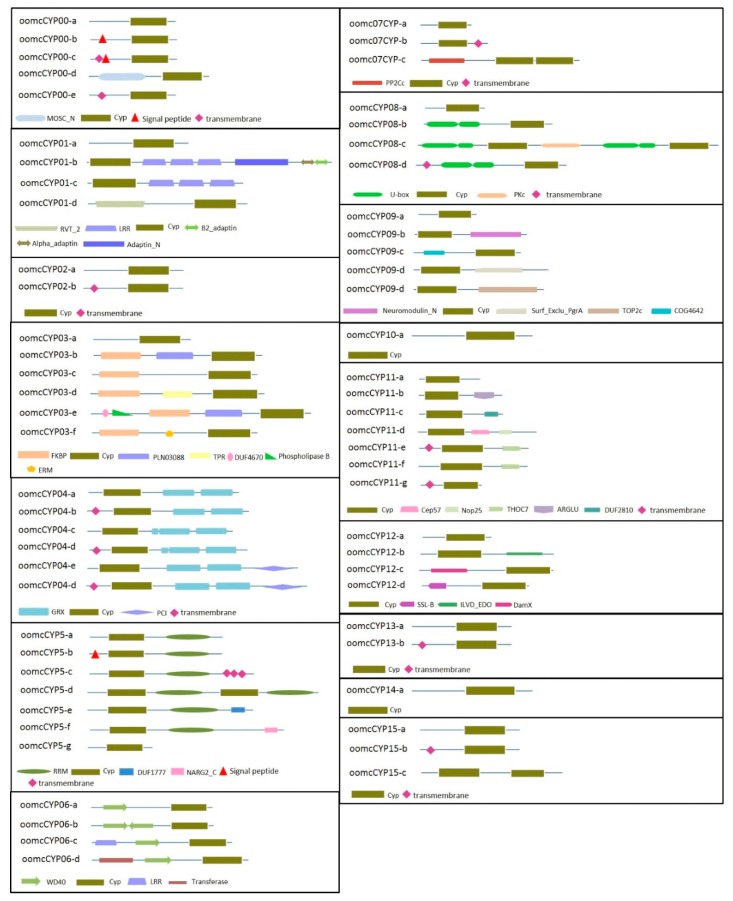
Domain architecture of CYPs in 21 oomycete species. CYPs are separated by orthogroup and further sub-dived by domain architecture. Key: CYP: cyclophilin; FKBP: FK506-binding proteins, LRR: leucine-rich repeat; TPR: tetracopeptide repeat; GRX: glutaredoxin; RRM: RNA recognition; WD40 repeat: potential functions include roles in signal transduction, pre-mRNA processing, and cytoskeleton assembly; U-box: modified DNA-binding ring-finger.

Cyclophilins in oomycetes were classified into two major categories: single-domain proteins (five orthogroups) and multi-domain proteins (10 orthogroups; Figure 1). Bigrams, defined as pairs of different domains in a protein, have been reported in eukaryotic species to enable coupling between two distinct cellular processes, and proteins enriched for bigrams may be involved in pathogenicity [25]. The overall number of bigrams in oomycetes was significantly higher than fungi but less than other species (e.g., *Drosophila melanogaster*) [25]. Investigating the CYP containing bigrams may therefore indicate the role these proteins play in oomycetes. Previously, six additional types of domains were reported in CYP proteins from *Phytophthora* spp.: a FK506-binding protein (FKBP) immunophilin domain, tetracopeptide repeat (TPR), glutaredoxin (GRX), RNA recognition (RRM), modified DNA-binding ring-finger (U-box), and WD40 repeat domains [17]. This study identified an additional 23 domains that formed bigrams with CYP domains. Five of the previously identified domain combinations were ubiquitous to all oomycetes: FKBP (PF00254; oomcCYP03), GRX (PF00462; oomcCYP04), RRM (PF00076; oomcCYP05), WD40 repeat (PF00400; oomcCYP06), and U-box (PF04564; oomcCYP08) domains [26,27,28,29,30]. FKBP-3TPR-CYP bigram has been reported to be present in unicellular eukaryotes, including ciliophora, oomycetes, diatoms, and dinoflagellates, and as inhibiting calcineurin (protein phosphatase 2B) in the presence of the cognate drugs to exhibit family-specific drug sensitivity [31,32]. This bigram was detected in other stramenopiles and alveolates, but not from Rhizaria, Plantae, or opisthokonts (Supplementary Figure S1). Other bigrams indicate a ubiquitous role in the oomycetes in detoxification, RNA recognition, protein–protein/protein–DNA interactions, and ubiquitination [33,34,35,36,37]. Therefore, CYPs may have a wide range of roles in oomycetes.

There was no strong evidence for secretion signals of all proteins in a single orthogroup, though a few proteins were implicated as being secreted (Supplementary Table S3). This does not preclude these proteins from being secreted or transported to the host through other mechanisms [38,39]. Additionally, secretion signals may be lost if the protein is incorrectly annotated with an early or late start codon predicted. Interestingly, 23 of 26 omcCYP04 proteins had a predicted transmembrane domain; oomcCYP02 proteins contained only a CYP domain, and omcCYP04 CYP proteins were bigrams with GRX (Figure 1; Supplementary Table S3).

### 2.2. Multidomain Analysis 

**Figure 2:**
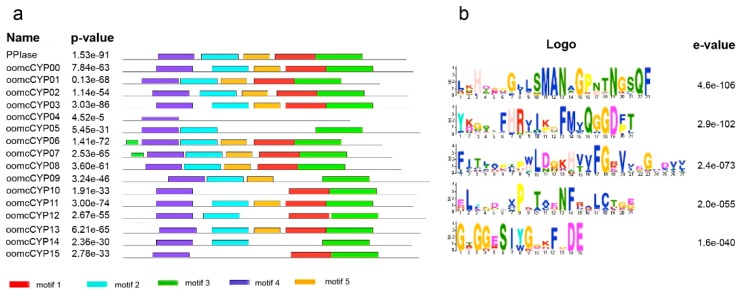
(**a**) Five conserved motifs were predicted in peptidyl-prolyl cis–trans isomerase (PPIase) and CYP consensus sequences from 16 orthogroups (from 22 oomycetes). (**b**) For PPIase and 16 oomcCYP consensus sequences in this comparison, five sites are highly conserved in the motifs.

**Figure 3:**
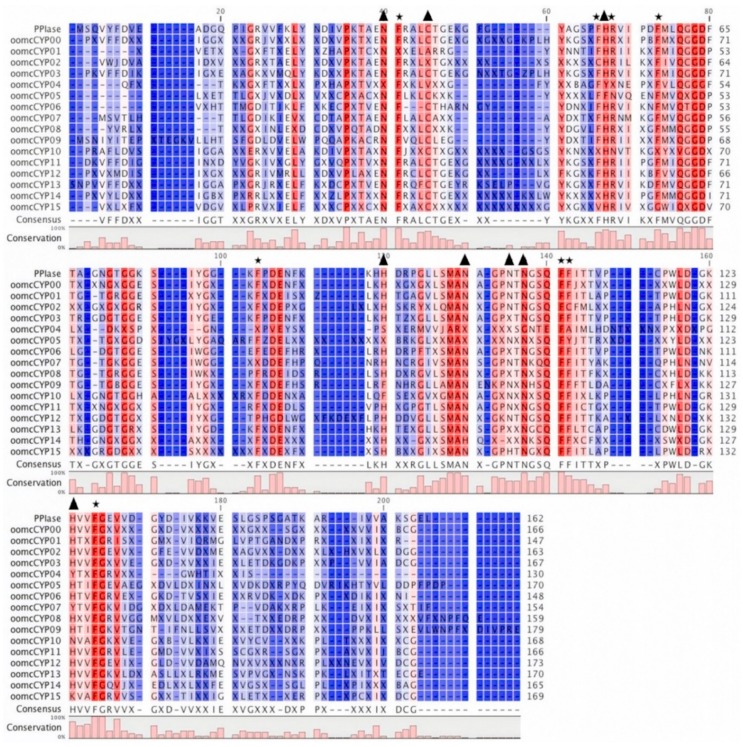
Alignment of PPIase and CYP domain consensus sequences of 16 orthogroups. Stars indicate residues conserved in plants, animals, and fungi, reportedly required for PPIase activity. Triangles indicate residues conserved in plants, animals, and fungi reportedly required for cyclosporin A (CsA) binding.

**Figure 4:**
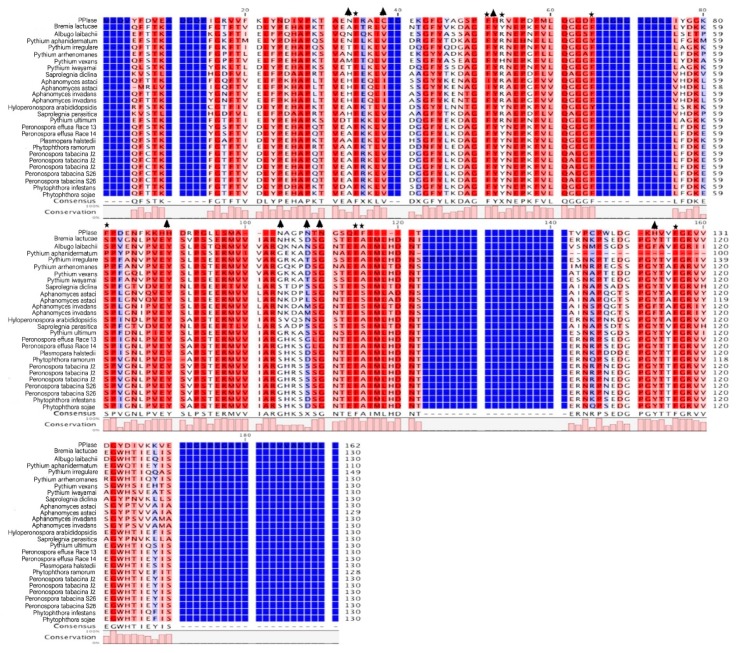
Alignment of PPIase and 16 oomcCYP04 CYP domain consensus sequences. Stars indicate residues conserved in plants, animals, and fungi, reportedly required for PPIase activity. Triangles indicate residues conserved in plants, animals, and fungi reportedly required for CsA binding. Dark blue columns indicate alignment positions where oomycete protein models are lacking residues compared to PPIase.

The CYP domains of oomycete species ranged from 121 to 259 residues in length. The consensus sequence for nine of the orthogroups contained five motif blocks (Figure 2) that were conserved in other eukaryotic CYP domains. The amino acid motifs QGGD and KHVVFG are associated with protein folding and stabilization in humans [40], and were present in the consensus sequence of 11 of the 16 orthogroups. The consensus sequence for all CYP orthogroups, except oomcCYP04, showed conservation of 65 to 130 residues dispersed across the CYP protein, including a CsA binding site and three conserved residues required for PPIase catalysis [41] (Supplementary Figure S2, Figure 3). When each ortholog of oomcCYP04 was aligned against PPIase, 55 residues were positionally conserved with the PPIase sequence. The annotated oomcCYP04 proteins were conserved across the oomycetes, although highly diverged from other orthogroups of oomycete cyclophilins (Figure 4).

### 2.3. Phylogenetics of CYPs in Oomycetes

**Figure 5:**
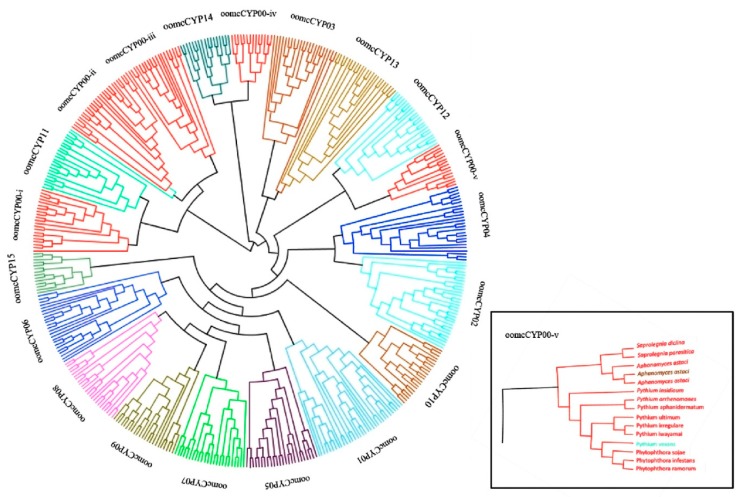
Phylogenetic relationships of oomcCYP domains. The phylogenetic tree was generated by RAxML, using amino acid sequences from 472 oomcCYP domains. Colors indicate the oomycete orthogroup. The inset shows a subtree of oomcCYP00-iv, which was not detected in the assemblies of downy mildews or *Albugo* spp.

The phylogenetic tree based on the CYP domain showed clustering correlated with orthology based on all-by-all protein alignments, though orthogroup oomcCYP00 was split into five clades (oomcCYP00-i, oomcCYP00-ii, oomcCYP00-iii, oomcCYP00-iv, and oomcCYP00-v) (Figure 5, Supplementary Table S4). OomcCYP00 was highly similar in all orthogroups (using *P. infestans* and *P. sojae* CYP sequence as the sample; Supplementary Table S4). As this was the largest orthogroup with proteins often only containing a single CYP domain (Figure 1), it is possible that multiple paralogs were assigned to a common orthogroup. Signal peptides or trans-membrane domains were often found encoded in proteins belonging to clade oomcCYP00-i. Although the majority of CYP domains clustered phylogenetically, there were some instances where clades containing CYP domains were assigned to different orthogroups (i.e., oomcCYP-v, Figure 5 inset). The phylogenetic analysis and annotations supported that downy mildew and *Albugo* species assemblies do not contain oomcCYP14 and oomcCYP15 cyclophilins. Additionally, two oomcCYP00 clades (oomcCYP00-iv and oomcCYP00-v) were not detected from these species (Figure 5). Like downy mildews, *Albugo* spp. are thought to have adapted to biotrophy from a non-biotrophic ancestor [42], meaning that these CYP proteins may have been lost from at least two lineages that independently adapted to biotrophy. If these proteins are not required for biotrophy, then a lack of selection and drift may have resulted in their loss. A similar conclusion was made for biotrophic downy mildews, which exhibited a depletion of pathogenicity as well as transporter and carbohydrate-associated domains, when compared to hemibiotrophs [43].

To study the relationship between oomycete CYPs and plant or fungal CYPs, the top 10 plant and fungal CYPs from National Center for Biotechnology Information (NCBI), ranked by percent identity, were added to the alignments, using *Phytophthora* proteins as queries. Phylogenetic trees were constructed from diverse oomycete, plant, and fungal CYP sequences. In most cases, oomycete orthogroups clustered together, away from plant and fungal CYP sequences (Supplementary Figure S3). This was not observed for oomcCYP01; proteins annotated in *Saprolegnia* and *Aphanomyces* species appeared closer to plant CPYs than other oomycete CYPs. Reciprocal BLAST [44] of *P. infestans* annotations supported orthology of oomycete proteins with plant and fungal proteins for CYPs belonging to oomcCYP00-iii, oomcCYP01, oomcCYP05, oomcCYP06, oomcCYP07, oomcCYP08, oomcCYP09, and oomcCYP13 (Supplementary Table S5). In addition, oomcCYP00-i, oomcCYP00-ii, oomcCYP03, and oomcCYP04 had the best reciprocal BLAST hits with one of either fungi or plants, but not both, supporting shared ancestry (Supplementary Table S5). Additional domains fused to CYP proteins may have resulted in the top hit identified being non-orthologous, such as for oomcCYP04, a CYP–GRX bigram (Figure 1). For the other eight *P. infestans* CYP proteins, the reciprocal BLAST hit for fungal and plant results was to other *P. infestans* CYP proteins (Supplementary Table S5). Only two of these eight had reciprocal BLAST hits when non-oomycete stramenopiles were surveyed (Supplementary Table S5). Therefore, these six protein lineages may be unique to the oomycetes. Interestingly, one of these lineages, oomcCYP14, was not detected in biotrophic oomycete species (Table 1).

### 2.4. Expression of CYPs in Different Life Stages of P. infestans and P. halstedii 

**Figure 6:**
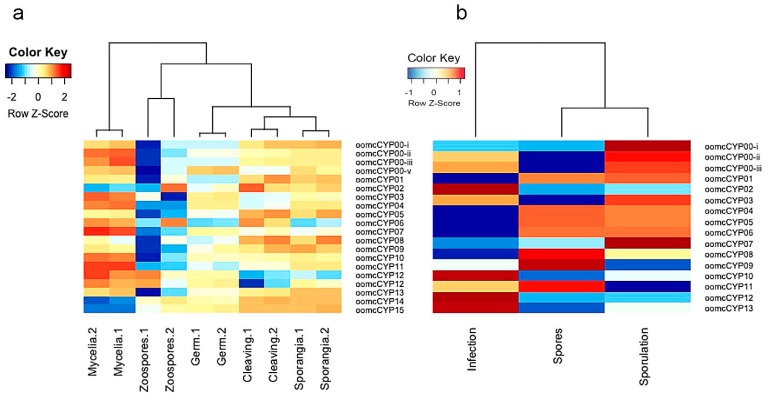
Expression analyses of (**a**) *P. infestans* and (**b**) *P. halstedii* CYP genes during their life cycles. The mRNA data was obtained from NCBI for the generation of heatmaps. The color scale in the heatmap indicates expression values; blue indicates low transcript abundance and red indicates high transcript abundance.

Expression of CYPs was characterized in different asexual life stages of the hemibiotroph *P. infestans* and the biotrophic *P. halstedii* (Figure 6). For *P. infestans*, life stage replicates clustered together, inferring a robust expression profile within biological replicates. The majority of CYPs were expressed in most life stages, except in zoospores, where the most variation between-replicates was observed (Figure 6). Generally, the highest CYP expression was detected in the mycelia time-point, where plant infection, including appressorium and haustoria formation, takes place [45]. Expression of oomcCYP14 and oomcCYP15 was highest in sporangia and slightly reduced in cleaving sporangia, zoospores, and germ tube forming time-points. Transcription of these genes was greatly reduced in the mycelia (Figure 6). OomcCYP01 and oomcCYP06 were upregulated in the cleaving sporangia stage. OomcCYP01 was identified as closely related to fungal PPIase-1 (Pin1) (e.g., XP_003177293.1 and KZZ96398.1; Supplementary Figure S3). Pin1 participates in the phosphorylation-dependent prolyl isomerization that changes the conformation of its substrates, thus controlling cell cycle progression in fungi [46].

The analysis of CYP expression during *P. halstedii* infection revealed that the expression profiles of CYPs between the infection time point (early stage of infection) and the spores time point were almost inverts of each other, except for oomcCYP00-i and oomcCYP07 (Figure 6b), indicating distinct, life stage-dependent expression of each orthologous group in *P. halstedii*. The sporulation and spore profiles were more similar to one another; the expression of oomcCYP01, oomcCYP04, oomcCYP05, and oomcCYP06 were very similar (Figure 6b). These proteins include CYP bigrams with GRX, RRM, and WD40 (Supplementary Table S3), indicating that these CYP proteins may be less important to establishing an infection. During infection, only oomcCYP02, oomcCYP10, oomcCYP12, and oomcCYP13 were highly expressed, consistent with a role in establishing infection. These proteins were not annotated as encoding additional domains, signal peptides, or transmembrane domains. In *P. halstedii,* cyclophilins phylogenetically linked to fungal Pin1 (oomcCYP01) had low expression levels during infection, but higher expression in spores, the opposite of what was observed in *P. infestans*. The difference between *P. halstedii* and *P. infestans* suggests that many cyclophilins may have opposite roles in the life-cycle for these two oomycetes.

The proteins absent in the biotrophic *P. halstedii* were poorly expressed in the mycelia of *P. infestans*, consistent with a non-critical role in infection. These proteins were commonly expressed in later stages of infection, including sporangia formation and cleavage. This expression pattern coincided with the necrotic stage of *P. infestans* infection, which was absent in downy mildews. The expression profile of these genes in *P. infestans* may be part of a transcription-level molecular signature for the onset of the hemibiotrophic phase. The absence of these genes in the genome of *P. halstedii* possibly reflects the lack of a selective pressure to maintain them during the evolution of its biotrophic life style.

## 3. Methods

### 3.1. Identification of CYPs from Oomycete Species

Oomycete genomes and annotations were downloaded from their respective sources (Table 2). InterProScan v5.33 [47] was run on the entire dataset and queried for proteins encoding a CYP domain (PF00160). Additional domains encoded in the same proteins were also identified. Signal peptide and transmembrane predictions were performed with SignalP4.0 [48] and TMHMM Server v2.0 [49], respectively. All annotations were run through OrthoFinder v2.2.1 [50] and queried for orthogroups containing CYPs. Consensus domain architecture for each orthogroup was defined and proteins that deviated from this consensus were subject to further manual inspection.

### 3.2. Multiple Sequence Alignment and Phylogenetic Analysis

Phylogenetics was used to investigate the evolutionary relationships among the oomycete CYPs. Coordinates for the CYP domains were obtained from searches against the NCBI conserved domain database [17] and InterProScan [47] using PF00160 to filter the latter [52]. The sequences were manually extracted. The protein sequences of the CYP domains were aligned using MAFFT v7.245 [53]. Consensus protein sequences were obtained from alignments using the CLC Genomics Workbench v 8.0.1 (https://www.qiagenbioinformatics.com; https://secure.clcbio.com/helpspot/index.php?pg=kb.page&id=78). Conserved amino acid motifs were identified using the MEME v5.0.5 suite (http://meme-suite.org/) [54] with default parameters (zero-ordered model of sequences, minimum width equal to 6 and maximum width equal to 50). *P. infestans* sequences were queried against the NCBI nucleotide (nt) database to independently identify cyclophilins of plants (taxid: 3193), animals (taxid: 33208), fungi (taxid: 4751), Rhizaria (taxid: 543769), Alveolata (taxid: 33630), and stramenopiles (taxid: 33634), using taxid numbers to reduce the database size. An additional search of stramenopiles was conducted excluding oomycetes (taxid: 4762). The top 10 non-redundant plant and fungal hits were aligned with each oomycete orthogroup. A maximum likelihood protein tree was produced using RAxML v8.2.9, with 1000 bootstraps and a GAMMA substitution model [55]. Alignments and trees were visualized using Geneious version R10 [56]. Reciprocal BLAST of the top fungal and plant hit was carried out against the *P. infestans* assembly to infer support for orthology.

### 3.3. Expression Analysis of Phytophthora infestans and Plasmopara halstedii 

Previously published transcriptome data of *P. infestans* and *P. halstedii* (SRR5179148 to SRR5179157 and ERR583683 to ERR583685) were used to investigate the transcription of CYPs at distinct asexual life stages. Reads were mapped to their respective assembly using STAR v2.6.0c (-quantMode GeneCounts) [57], trimmed means of M normalization was applied to the mapped reads [58,59], and they were analyzed in RStudio [60]. Heatmaps for life stage specificity of the expression of CYP proteins of *P. infestans* and *P. halstedii* were generated in RStudio using tidyverse, ggplot2 [61], gplots [62], and edgeR [63].

## 4. Conclusions

We conducted a comprehensive sequence analysis of the CYPs encoded in the genome assemblies of 23 oomycetes, from 21 species. The oomycete CYPs were clustered into 16 orthogroups, largely supported by phylogenetic analysis of the CYP domains. Six CYP orthogroups included proteins that formed bigrams with a diverse range of domains indicative of a wide diversity of functions, which may include virulence. Significantly, the CYP-FBKP bigram (oomcCYP03) was found to be unique to stramenopiles and alveolates, and was not detected in Rhizaria, Plantae, or Opisthokonta. The function of these proteins is yet to be elucidated. Variable transcription of every CYP encoded by the hemibiotroph *P. infestans* and the biotroph *P. halstedii* was detected at different times throughout the course of infection. The differential expression of CYPs during an infection cycle in these oomycetes is consistent with CYPs playing diverse functions including, but not exclusively, pathogenicity.

## Figures and Tables

**Table 1:** Summary of the cyclophilin (CYP) protein sequences from 23 oomycete assemblies (21 species).

Species Name	Obligate Biotroph?	CYP Proteins	CYP Domains Predicted Complete	oomcCYP00	oomcCYP01	oomcCYP02	oomcCYP03	oomcCYP04	oomcCYP05	oomcCYP06	oomcCYP07	oomcCYP08	oomcCYP09	oomcCYP10	oomcCYP11	oomcCYP12	oomcCYP13	oomcCYP14	oomcCYP15
*Albugo candida*	+	15	14	2	1	1	2	0	2	1	1	1	1	1	1	0	1	0	0
*Albugo laibachii*	+	15	15	2	2	1	1	1	1	1	1	1	1	1	2	0	0	0	0
*Aphanomyces astaci*	-	35	30	6	8	2	2	2	1	1	1	1	2	1	1	1	1	3	2
*Aphanomyces invadans*	-	33	30	7	1	6	2	2	1	3	2	2	1	1	1	1	1	1	1
*Bremia lactucae*	+	18	15	5	1	1	1	1	1	1	1	1	1	1	1	1	1	0	0
*Hyaloperonospora arabidopsidis*	+	15	12	4	1	1	1	1	0	1	0	1	1	1	1	1	1	0	0
*Peronospora effusa Race 13*	+	16	14	3	1	1	1	1	1	1	1	1	1	1	1	1	1	0	0
*Peronospora effusa Race 14*	+	16	15	3	1	1	1	1	1	1	1	1	1	1	1	1	1	0	0
*Peronospora tabacina J2*	+	28	20	5	3	2	1	3	1	1	3	2	1	1	1	2	2	0	0
*Peron o spora tabacina S26*	+	28	25	6	2	1	2	2	3	1	2	2	1	1	1	2	2	0	0
*Plasmopara halstedii*	+	16	15	3	1	1	1	1	1	1	1	1	1	1	1	1	1	0	0
*Phytophthora infestans*	-	20	20	4	1	1	1	1	1	1	1	1	1	1	1	2	1	1	1
*Phytophthora sojae*	-	19	19	5	1	1	1	1	1	1	1	1	1	1	1	1	1	1	0
*Phytophthora ramorum*	-	20	19	5	1	1	1	1	1	1	1	0	1	1	1	1	1	2	1
*Pythium aphanidermatum*	-	20	17	5	1	1	1	1	1	1	1	1	1	1	1	1	1	1	1
*Pythium arrhenomanes*	-	22	18	6	1	1	1	1	1	1	1	1	2	1	1	1	1	1	1
*Pythium insidiosum*	-	13	12	3	1	1	1	0	1	1	1	1	0	1	0	0	0	1	1
*Pythium irregulare*	-	20	20	5	1	1	1	1	1	1	1	1	1	1	1	1	1	1	1
*Pythium iwayam a i*	-	20	17	5	1	1	1	1	1	1	1	1	1	1	1	1	1	1	1
*Pythium ultimum*	-	20	18	5	1	1	1	1	1	1	1	1	1	1	1	1	1	1	1
*Pythium vexans*	-	20	18	4	2	1	1	1	1	1	1	1	1	1	1	1	1	1	1
*Saprolegnia diclina*	-	21	21	6	1	1	1	1	1	1	1	1	1	1	1	1	1	1	1
*Saprolegnia parasitica*	-	22	22	6	1	2	1	1	1	1	1	1	1	1	1	1	1	1	1

**Table 2:** Published, annotated oomycete draft genome assemblies used in this study.

Species	Link/Reference
*Albugo candida*	http://protists.ensembl.org/Albugo_candida/Info/Index
*Albugo laibachii*	http://protists.ensembl.org/Albugo_laibachii/Info/Index
*Aphanomyces astaci*	http://protists.ensembl.org/Aphanomyces_astaci/Info/Index
*Aphanomyces invadans*	http://protists.ensembl.org/Aphanomyces_invadans/Info/Index
*Bremia lactucae*	https://www.ncbi.nlm.nih.gov/assembly/GCA_004359215.1/
*Hyaloperonospora arabidopsidis*	http://protists.ensembl.org/Hyaloperonospora_arabidopsidis/Info/Index
*Peronospora effusa Race 13*	https://www.ncbi.nlm.nih.gov/assembly/GCA_003843895.1
*Peronospora effusa Race 14*	https://www.ncbi.nlm.nih.gov/assembly/GCA_003704535.1
*Peronospora tabacina J2*	[51]
*Peronospora tabacina S26*	[51]
*Plasmopara halstedii*	http://protists.ensembl.org/Plasmopara_halstedii/Info/Index
*Phytophthora infestans*	http://protists.ensembl.org/Phytophthora_infestans/Info/Index
*Phytophthora sojae*	http://protists.ensembl.org/Phytophthora_sojae/Info/Index
*Phytophthora ramorum*	http://protists.ensembl.org/Phytophthora_ramorum/Info/Index
*Pythium aphanidermatum*	http://protists.ensembl.org/Pythium_aphanidermatum/Info/Index
*Pythium arrhenomanes*	http://protists.ensembl.org/Pythium_arrhenomanes/Info/Index
*Pythium irregulare*	http://protists.ensembl.org/Pythium_irregulare/Info/Index
*Pythium iwayamai*	http://protists.ensembl.org/Pythium_iwayamai/Info/Index
*Pythium insidiosum*	http://protists.ensembl.org/Pythium_inhsidiosum/Info/Index
*Pythium ultimum*	http://protists.ensembl.org/Pythium_ultimum/Info/Index
*Pythium vexans*	http://protists.ensembl.org/Pythium_vexans/Info/Index
*Saprolegnia diclina*	http://protists.ensembl.org/Saprolegnia_diclina_vs20/Info/Index
*Saprolegnia parasitica*	http://protists.ensembl.org/Saprolegnia_parasitica_cbs_223_65/Info/Index

## Supplementary Materials

The following are available online at https://www.mdpi.com/2076-0817/9/1/24/s1: Supplementary Figure S1: The CYP bigram present in stramenopiles and alveolates but undetected in Rhizaria. Supplementary Figure S2: Alignment of human PPIase and CYPs. Supplementary Figure S3: Study the relationship between oomycete CYPs and plant or fungal CYPs. Supplementary Table S1: All sequence names of 23 oomycete assemblies in 16 orthogroups. Supplementary Table S2: List of misannotated proteins. Supplementary Table S3: The count of functional domains detected in the structure analysis. Supplementary Table S4: The identity of all CYP sequences, explaining that oomcCYP00-i to oomcCYP00-v belong to one orthogroup (the black box is oomcCYP00). Supplementary Table S5: Reciprocal protein BLAST (BLASTp) results.
