# Supplementary figures and images for: Genome-Wide Analysis of Cyclophilin Proteins in 21 Oomycetes
Source: Pathogens. 2019 Dec 26;9(1):24. doi: 10.3390/pathogens9010024 (PMC7168621; doi:10.3390/pathogens9010024)

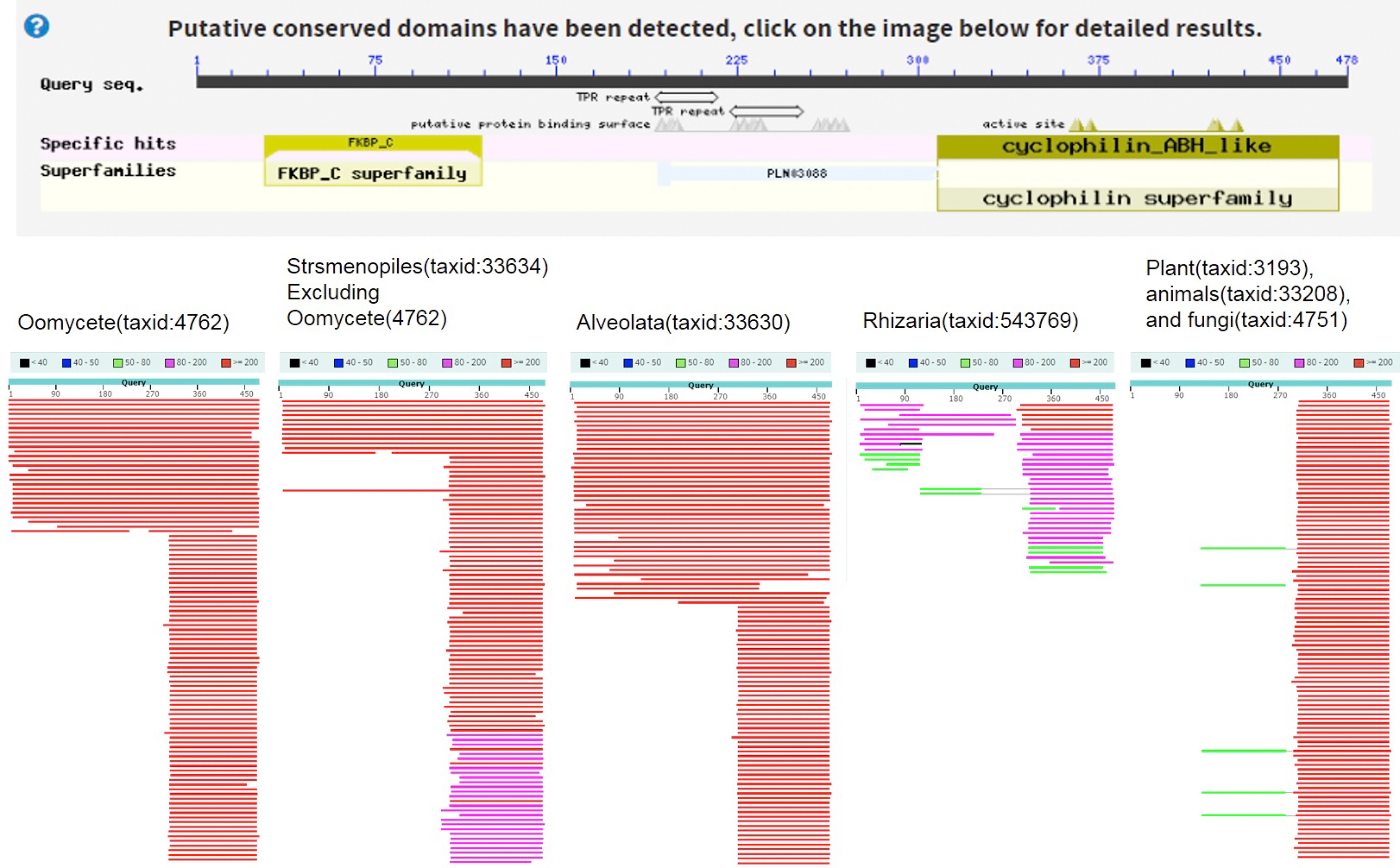

Supplement: Supplementary file 1 [file pathogens-09-00024-s001.zip › pathogens-665006-SI/Supplementary Figure S1.jpg]

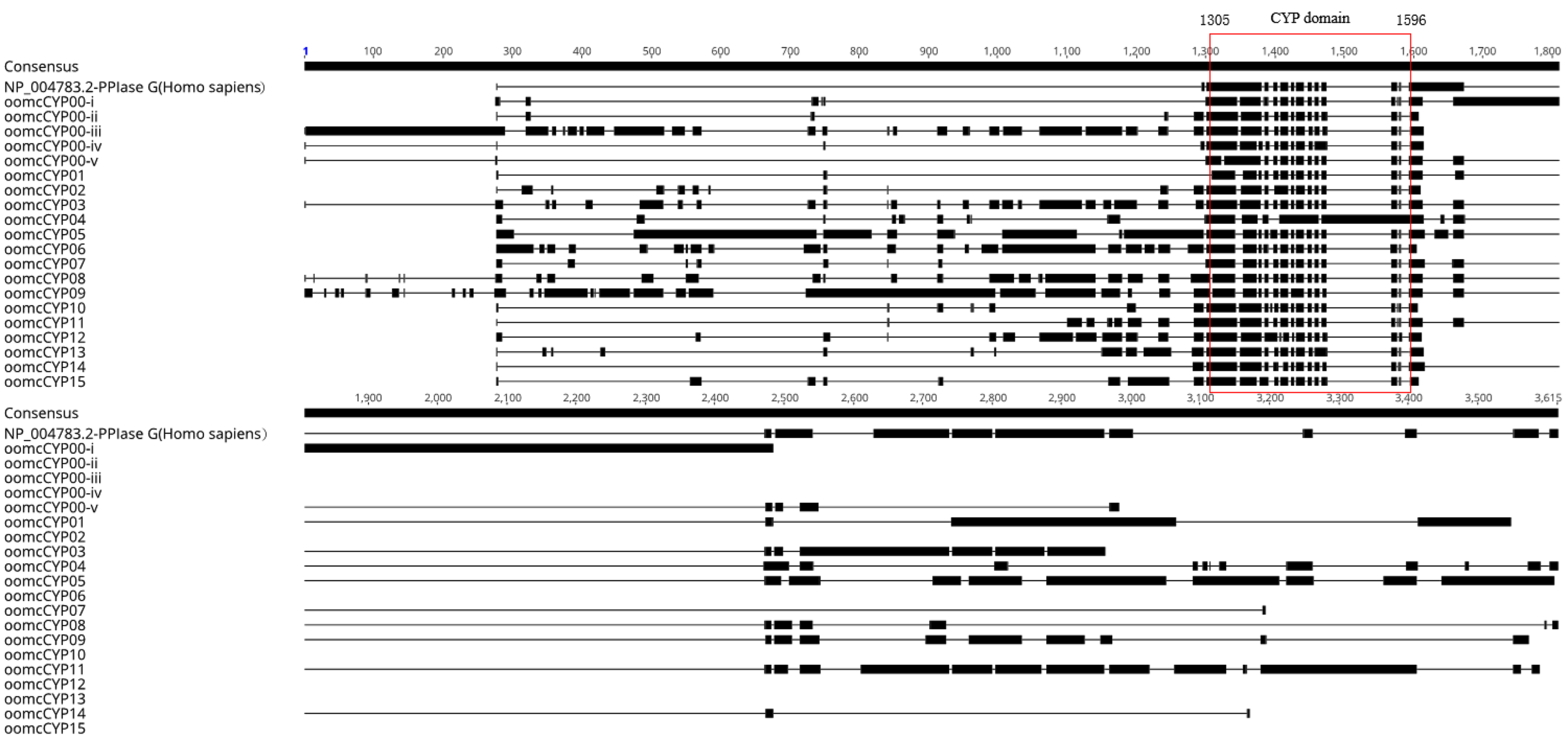

Supplement: Supplementary file 1 [file pathogens-09-00024-s001.zip › pathogens-665006-SI/Supplementary Figure S2.jpg]
